# Adverse events of the thyroid peroxidase inhibitor methimazole in the treatment of hyperthyroidism: a comprehensive analysis from the first quarter of 2004 to the first quarter of 2025

**DOI:** 10.3389/fendo.2025.1680281

**Published:** 2025-10-01

**Authors:** Yixin Sun, Shuai Wang, Xu Zhou

**Affiliations:** ^1^ School of Medicine, Xiamen University, Xiamen, Fujian, China; ^2^ National Institute for Data Science in Health and Medicine, School of Medicine, Xiamen University, Xiamen, Fujian, China; ^3^ Department of Minimally Invasive and Interventional Oncology, Zhongshan Hospital of Xiamen University, School of Medicine, Xiamen University, Xiamen, Fujian, China

**Keywords:** methimazole, pharmacovigilance, FAERS, ROR, BCPNN, EBGM

## Abstract

**Purpose:**

The primary objective of this study is to systematically monitor and comprehensively characterize adverse events (AEs) associated with methimazole, which is utilized in the treatment of hyperthyroidism.

**Method:**

A comprehensive analysis of the US FDA Adverse Event Reporting System (FAERS) database was conducted, covering data from the first quarter of 2004 to the first quarter of 2025. Multiple signal detection algorithms, including the ROR, PRR, BCPNN, and EBGM, were employed for conduct disproportionality analysis to efficiently mine the data and accurately identify signals associated with AEs related to methimazole.

**Result:**

After analyzing 1,908 patient cases with 6,449 reported AEs linked to methimazole, the study confirmed AEs like agranulocytosis, pyrexia, hypothyroidism, and drug-induced liver injury aligning with the drug’s package insert. Interestingly, several previously unreported AEs, such as premature baby, polyarthritis, pleural effusion, septic shock, cholestasis and jaundice cholestatic were identified. The findings indicate potential unrecognized AEs and highlight the importance of continued pharmacovigilance and rigorous drug safety surveillance. The median onset time of methimazole related AEs was 27 days (interquartile range [IQR]: 5–58 days), indicating that the majority of cases occurred early after initiating use methimazole.

**Conclusion:**

This study confirms that agranulocytosis, pyrexia, exposure during pregnancy, and hyperthyroidism are AEs associated with methimazole use. It also identifies new safety signals, including premature infants, polyarthritis, pleural effusion, septic shock, cholestasis, and cholestatic jaundice, that warrant further investigation. These findings support deeper analysis of the relationship between methimazole and adverse events and may help improve patient safety and clinical outcomes.

## 1 Introduction

Hyperthyroidism, commonly known as “thyroid toxicity”, is a complex endocrine disorder characterized by excessive secretion of thyroid hormones, leading to an abnormally elevated metabolic rate (1). The global prevalence of this condition ranges from 0.2% to 1.3%, with the condition affecting individuals of all age groups but occurring more frequently in women between 20 and 50 years of age (2). The etiology of hyperthyroidism is heterogeneous, and its clinical manifestations vary widely (3). Its main characteristic is that excessive thyroid hormones (mainly T3 and T4) synthesized and secreted by the thyroid gland enter the bloodstream, resulting in a significant increase in the metabolic level of body tissues. This leads to an accelerated metabolism of the body and triggers a series of clinical symptoms (4–8). Among the various causes leading to hyperthyroidism, Graves’ disease accounts for the majority of cases. Global statistics show that the prevalence of Graves’ disease among women is approximately 2%, and among men it is about 0.5%. Patients typically exhibit elevated levels of triiodothyronine (T3) and/or free thyroxine (FT4), which affects approximately 0.2% to 1.4% of the global population. Additionally, there is a condition known as subclinical hyperthyroidism, characterized by a lower concentration of thyroid stimulating hormone (TSH), while the levels of T3 and FT4 remain within the normal range (9, 10). Unlike overt hyperthyroidism, the clinical symptoms of subclinical hyperthyroidism are often atypical or lack obvious manifestations. Patients are usually discovered incidentally during routine physical examinations or when seeking treatment for other diseases. Although its clinical manifestations are concealed, multiple studies have suggested that the continuous inhibition of TSH is closely related to an increased risk of cardiovascular events, atrial fibrillation, osteoporosis, and an increased incidence of fractures, especially in the elderly population (11–13). Hyperthyroidism not only has a multifaceted negative impact on patients’ physical health but can also lead to a range of serious complications. Therefore, accurate diagnosis and the formulation of individualized treatment strategies are essential for improving patient outcomes, with early identification and timely intervention being particularly critical. Therefore, the early identification and intervention of subclinical hyperthyroidism has become one of the focuses of attention in the field of endocrinology. As an anti-thyroid drug widely used in the current market, methimazole has received a lot of research attention. In recent years, studies on the clinical efficacy and safety of methimazole, especially the detection of adverse events, have become an important direction in the evaluation of drug safety. This study, based on real-world data, conducted a systematic analysis of adverse events related to the use of methimazole. It also integrated four algorithms. The research results are helpful for a comprehensive assessment of its safety characteristics and provide a scientific basis for the formulation and management of individualized clinical medication strategies.

The primary mechanism of action of methimazole is to inhibit thyroid peroxidase (TPO), which is a rate-limiting enzyme that plays a crucial role in the synthesis of thyroid hormones and can regulate the synthesis of thyroid hormones production (14). TPO catalyzes the iodination of tyrosine residues on the thyroid globulin and the subsequent coupling reactions between these tyrosine residues. These processes are crucial for the production of thyroid hormones. Methimazole exerts an anti-thyroid hormone synthesis effect by binding to TPO, hindering the iodination and coupling reactions of tyrosine residues, thereby reducing the production of thyroid hormones and exerting an anti-thyroid effect (15).

Methimazole is a common anti-thyroid drug. It blocks the making of thyroid hormones. This can help reduce bone loss caused by thyrotoxicosis. But the drug can cause side effects. In a Danish study at many centers (n=208; average treatment 22 months; range 0.5–49 months), 10% of patients had drug-related side effects. Seventy-five percent happened in the first 6 months. By 24 months, when the dose was 5 mg/day, no new side effects were seen in that group. The most common side effects were skin problems (68%) (16). A clinic study in Brazil found that many adults taking methimazole had a positive ANCA blood test (17).These results show that close follow-up is needed early in treatment, especially in the first 6 months. Watch for common problems such as skin reactions.

These findings show that close monitoring is needed, especially in the first 6 months. Watch for common side effects, such as skin problems. The FDA Adverse Event Reporting System (FAERS) is one of the world’s largest pharmacovigilance databases for adverse drug events, providing a wealth of data for the study of drug safety (18–21). We analyzed the data in the FAERS database using four algorithms, namely ROR (22), PRR (23), BCPNN (24) and MGPS. The PTs that met the criteria of all four algorithms were included in our subsequent study. By leveraging the extensive data resources of the FAERS database and the precise analytical capabilities of signal detection techniques, we are able to conduct a comprehensive and in-depth evaluation of the AEs associated with methimazole. This provides clinical physicians with detailed safety information for reference when using this drug. This helps clinical physicians make more accurate decisions when weighing the efficacy and safety of drugs, reduces the risk of adverse drug events, ensures patient medication safety, and thereby enhances the overall effectiveness and quality of clinical treatment, promoting the continuous improvement of rational drug use in clinical practice.

## 2 Methods

### 2.1 Data collection

The FAERS is a publicly accessible database that primarily supports post-marketing surveillance programs for drugs and therapeutic biologics. It covers all adverse event and medication error information directly obtained from patients and healthcare practitioners. In this study, the real-world data related to AEs of methimazole from the first quarter of 2004 to the first quarter of 2025 were downloaded as raw ASCII data packages and imported into SAS 9.4 software for data mining and statistical analysis. After data download, data cleaning was performed, including removing duplicates, deleting missing data, and excluding withdrawn or deleted reports. The adverse event names in the FAERS database were coded using the latest MedDRA dictionary (MedDRA 28.0). According to the hierarchical structure of MedDRA 28.0 terminology, significant AEs were mapped to PT and System Organ Classes (SOC). Based on data from the FAERS database, we conducted differential and subgroup analyses to provide a comprehensive assessment of the distribution and characteristics of methimazole-associated AEs across population groups and clinical strata.

### 2.2 Statistical analysis

**Figure 1:**
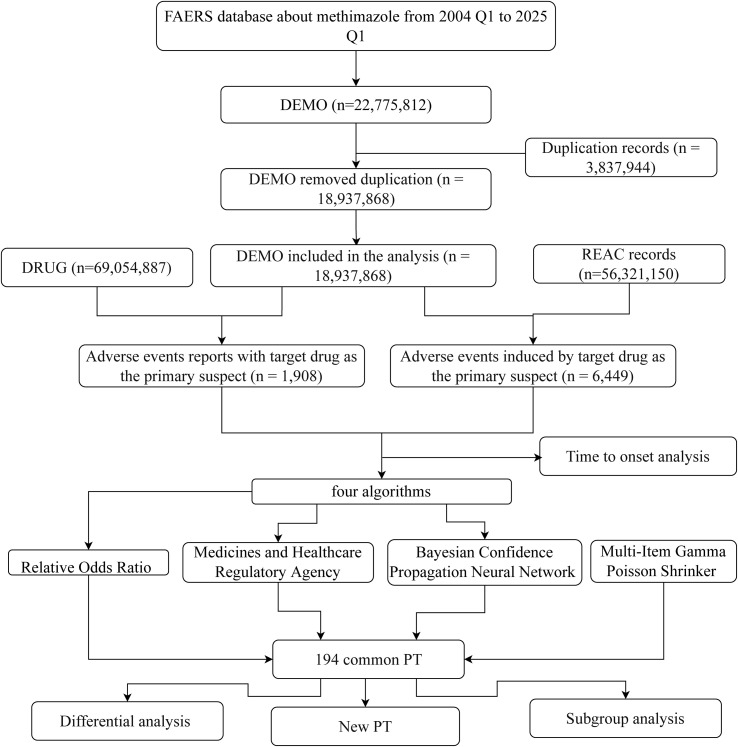
The flowchart of the research in this manuscript.

Patients may experience AEs after using methimazole. To look for possible links between methimazole and these AEs, we used four disproportionality methods (25): ROR, PRR, BCPNN, and MGPS. To identify potential safety signals, we applied four disproportionality methods. The Reporting Odds Ratio (ROR) measures how often a specific adverse event (AE) is reported with methimazole compared to other drugs, while the Proportional Reporting Ratio (PRR) assesses the proportion of methimazole-related reports that include a given AE relative to other drugs. In addition, two Bayesian approaches were used: the Bayesian Confidence Propagation Neural Network (BCPNN), which calculates an Information Component (IC) to quantify the strength of drug–AE associations, and the Empirical Bayes Geometric Mean (EBGM), which adjusts for small sample sizes to provide more stable estimates and avoid overestimating rare events. With four carefully selected methods, we systematically compared the adverse event reporting rates of methimazole in the FAERS database. These methods quantified the signal strength through the standardized formulas and thresholds detailed in **Supplementary Table 1**, providing highly convincing evidence for the safety assessment of methimazole and ensuring the scientific and reliable nature of the assessment results. The flowchart of this study is presented in **Figure 1**.

## 3 Result

### 3.1 Demographic statistics of related to methimazole in the FAERS database

**Figure 2:**
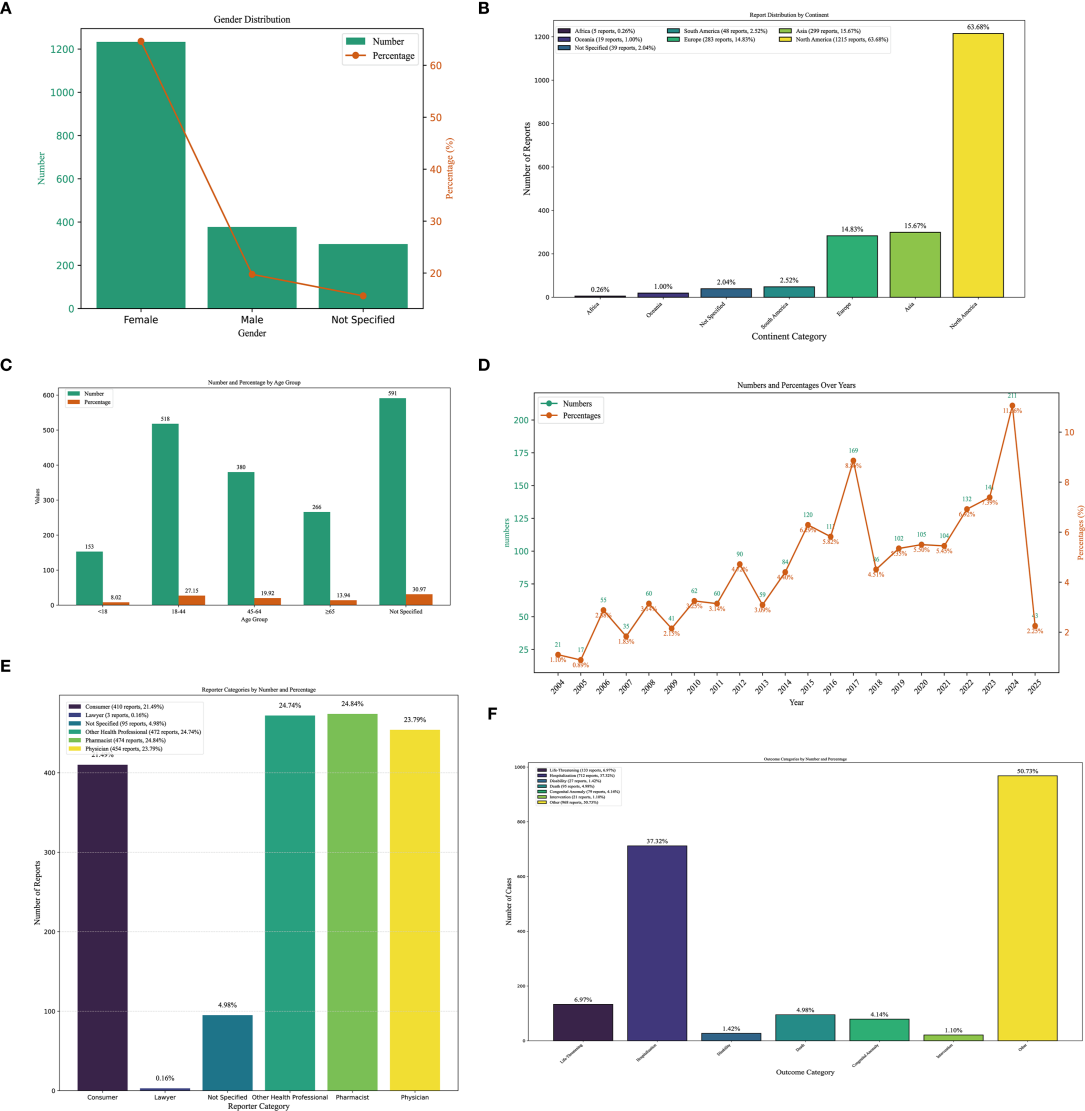
Clinical characteristics of methimazole-associated reports from the FAERS database. **(A)** Gender. **(B)** Continent. **(C)** The occurrence time of adverse events after using methimazole. **(D)** The frequency of AEs occurring each year from 2004 to 2025. **(E)** reporter. **(F)** outcomes.

Interrogation of the FAERS database identified 1,908 patients reporting methimazole-associated AEs, accounting for 6,449 individual events overall. Each patient experienced an average of 2.4 AEs. The gender distribution analysis revealed that the proportion of AEs in females (64.62%, n = 1,233), whereas males accounted for 19.76% (n=377) (**Figure 2A**). The ratio of the female population number to the male population number is 3.27. Geographically, reports originating from North America (n = 1,215; 63.68%) and Asia (n = 299; 15.67%) accounted for -a substantially greater number of AEs compared with other continents (**Figure 2B**). Age-stratified analysis showed that, excluding patients with unknown age, the largest proportion of AEs occurred in the 18–44-year age group (27.15%, n = 518), followed by the 45–64-year group (19.92%, n = 380), those ≥65 years (13.94%, n = 266), and patients younger than 18 years (8.02%, n = 153). The overall mean age was 45.06 ± 20.59 years (**Figure 2C**). Temporal trends indicated that the number of reported AEs peaked in 2024 (n = 211), with the second highest in 2017 (n = 169, 8.86%). Overall, the annual trend showed an initial increase, followed by a decline, and then a renewed increase (**Figure 2D**). In the stratified analysis by reporter type, pharmacists accounted for the largest proportion of AE reports (24.84%, n = 474), whereas lawyers contributed the smallest fraction (**Figure 2E**). Regarding outcomes, the most frequent category was “other” (50.73%, n = 968), followed by hospitalization (initial or prolonged) (37.32%, n = 712) and life-threatening events (6.97%, n = 133) (**Figure 2F** **) (** **Table 1**).

**Table 1:** Characteristics of AEs reports about methimazole.

Characteristics	Number (Percentage%)
Sex	
Female	1233(64.62)
Male	377(19.76)
Not Specified	298(15.62)
Age(years)	
<18	153( 8.02)
18-44	518(27.15)
45-64	380(19.92)
≥65	266(13.94)
NotSpecified	591(30.97)
Age(years)	
N(Missing)	1317(591)
Mean(SD)	45.06(20.59)
Median(Q1,Q3)	44.00(30.00,61.00)
Min,Max	0.00,94.00
Report year	
2004	21( 1.10)
2005	17( 0.89)
2006	55( 2.88)
2007	35( 1.83)
2008	60( 3.14)
2009	41( 2.15)
2010	62( 3.25)
2011	60( 3.14)
2012	90( 4.72)
2013	59( 3.09)
2014	84( 4.40)
2015	120( 6.29)
2016	111( 5.82)
2017	169( 8.86)
2018	86( 4.51)
2019	102( 5.35)
2020	105( 5.50)
2021	104( 5.45)
2022	132( 6.92)
2023	141( 7.39)
2024	211(11.06)
2025	43( 2.25)
Reporter	
Consumer	410(21.49)
Lawyer	3( 0.16)
Not Specified	95( 4.98)
Other health-professional	472(24.74)
Pharmacist	474(24.84)
Physician	454(23.79)
Outcomes	
Life-Threatening	133( 6.97)
Hospitalization - Initial or Prolonged	712(37.32)
Disability	27( 1.42)
Death	95( 4.98)
Congenital Anomaly	79( 4.14)
Required Intervention to Prevent Permanent	21( 1.10)
Impairment/Damage	
Other	968(50.73)
Adverse event occurrence time(days)	
0-30d	144( 7.55)
31-60d	50( 2.62)
61-90d	16( 0.84)
91-120d	5( 0.26)
121-150d	4( 0.21)
151-180d	5( 0.26)
181-360d	11( 0.58)
>360d	22( 1.15)
Missing or outlier(less than 0)(%)	1651(86.53)
Adverse event occurrence time(days)	
N(Missing)	257(1651)
Mean(SD)	164.67(538.39)
Median(Q1,Q3)	27.00(5.00,58.00)
Min,Max	0.00,4414.00
Weight(kg)	
N(Missing)	356(1552)
Mean(SD)	61.75(29.20)
Median(Q1,Q3)	63.70(51.73,76.20)
Min,Max	1.38,208.00

### 3.2 Signal detection of methimazole-related adverse events at the SOC level using disproportionality analysis in the FAERS database

**Figure 3:**
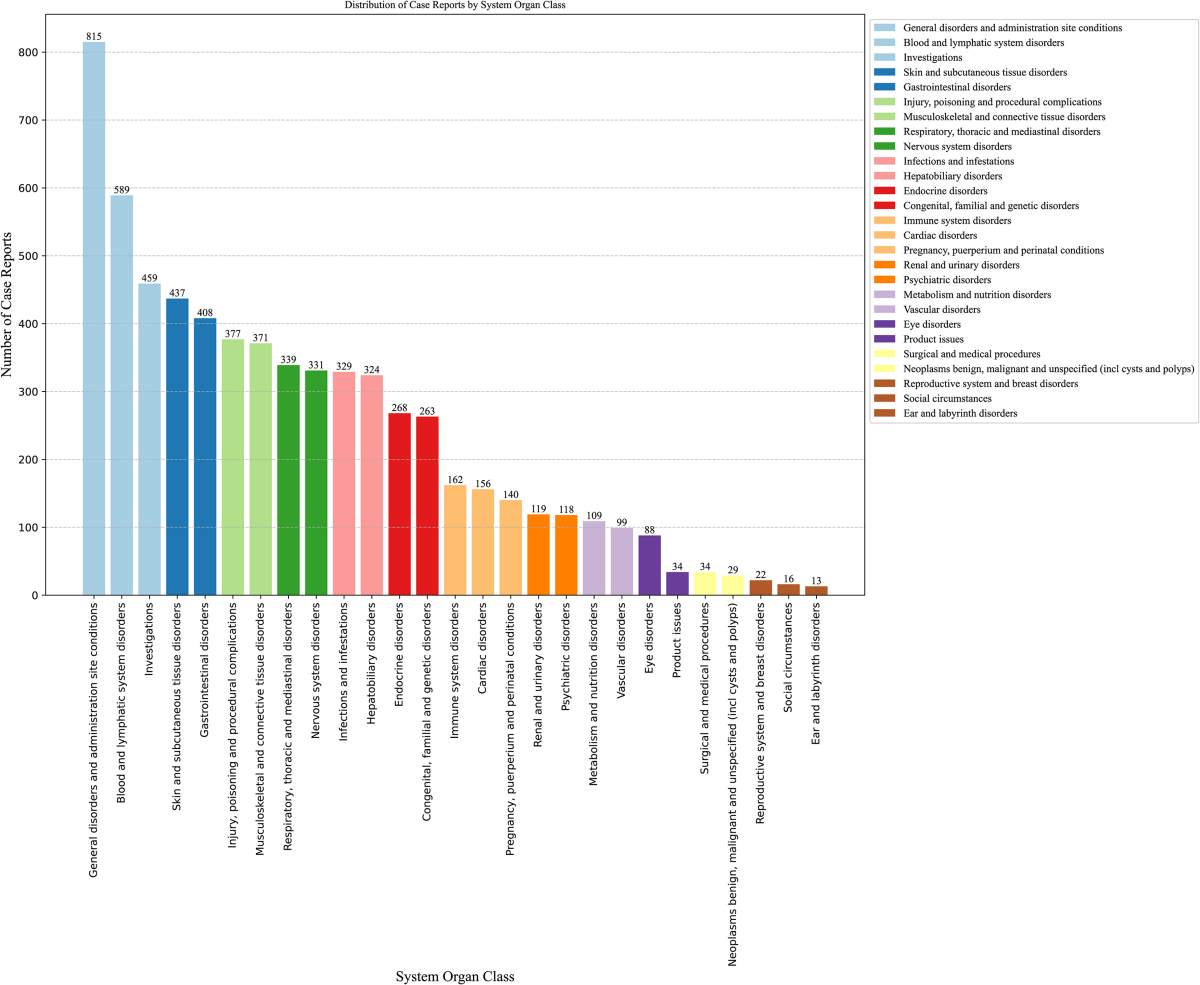
Signal detection related to SOC levels about methimazole (ranking based on number of reports).

The number of AEs reported for thiamazole in different SOCs is presented in **Table 2** **and** **Figure 3**. We identified a total of 27 AEs related to the system organs. These results are ranked by frequency of case reports, and those with the lower limit of the 95% ROR confidence interval being less than 1 are excluded. Ranked by the number of case reports, the six most frequently reported SOCs were blood and lymphatic system disorders (n=589), investigations (n=459), skin and subcutaneous tissue disorders (n=437), musculoskeletal and connective tissue disorders (n=371), respiratory, thoracic and mediastinal disorders (n=339), and hepatobiliary disorders (n=324).Ranked by the number of ROR, the six most frequently reported SOCs were Endocrine disorders (ROR = 16.97), Congenital, familial and genetic disorders (ROR = 14.19), Blood and lymphatic system disorders (ROR = 5.86), Hepatobiliary disorders (ROR = 5.70), Pregnancy, puerperium and perinatal conditions (ROR = 5.19), and Immune system disorders (ROR = 2.31).

**Table 2:** The signal intensity of ADEs related to methimazole at the system organ category (SOC) level in the FAERS database.

System organ Class (SOC)	SOC code	Case reports	ROR (95% CI)	PRR (95% CI)	Chi_square	IC (IC025)	EBGM (EBGM05)
General disorders and administration site conditions	10018065	815	0.68 (0.64,0.74)	0.72 (0.68,0.77)	103.28	-0.46 (-0.57)	0.72 (0.67)
Blood and lymphatic system disorders	10005329	589	5.86 (5.38,6.37)	5.41 (5.01,5.85)	2153.97	2.44 (2.30)	5.41 (4.97)
Investigations	10022891	459	1.18 (1.07,1.29)	1.16 (1.06,1.27)	11.12	0.22 (0.08)	1.16 (1.06)
Skin and subcutaneous tissue disorders	10040785	437	1.27 (1.15,1.40)	1.25 (1.14,1.37)	23.54	0.32 (0.18)	1.25 (1.14)
Gastrointestinal disorders	10017947	408	0.73 (0.66,0.80)	0.74 (0.68,0.82)	39.07	-0.43 (-0.57)	0.74 (0.67)
Injury, poisoning and procedural complications	10022117	377	0.53 (0.48,0.59)	0.56 (0.51,0.62)	146.74	-0.84 (-0.99)	0.56 (0.50)
Musculoskeletal and connective tissue disorders	10028395	371	1.12 (1.01,1.25)	1.11 (1.01,1.23)	4.57	0.16 (0.00)	1.11 (1.00)
Respiratory, thoracic and mediastinal disorders	10038738	339	1.12 (1.01,1.25)	1.12 (1.01,1.24)	4.37	0.16 (-0.00)	1.12 (1.00)
Nervous system disorders	10029205	331	0.59 (0.52,0.65)	0.61 (0.55,0.67)	91.99	-0.72 (-0.88)	0.61 (0.54)
Infections and infestations	10021881	329	0.97 (0.87,1.08)	0.97 (0.87,1.08)	0.35	-0.05 (-0.21)	0.97 (0.87)
Hepatobiliary disorders	10019805	324	5.70 (5.10,6.38)	5.47 (4.92,6.08)	1193.19	2.45 (2.27)	5.47 (4.89)
Endocrine disorders	10014698	268	16.97 (15.01,19.18)	16.31 (14.50,18.34)	3853.21	4.02 (3.77)	16.28 (14.40)
Congenital, familial and genetic disorders	10010331	263	14.19 (12.54,16.06)	13.66 (12.13,15.37)	3089.04	3.77 (3.52)	13.64 (12.05)
Immune system disorders	10021428	162	2.31 (1.98,2.70)	2.28 (1.96,2.65)	117.26	1.19 (0.95)	2.28 (1.95)
Cardiac disorders	10007541	156	0.92 (0.79,1.08)	0.93 (0.79,1.08)	0.96	-0.11 (-0.34)	0.93 (0.79)
Pregnancy, puerperium and perinatal conditions	10036585	140	5.19 (4.39,6.13)	5.10 (4.33,6.01)	462.85	2.35 (2.06)	5.10 (4.31)
Renal and urinary disorders	10038359	119	0.97 (0.81,1.17)	0.97 (0.82,1.16)	0.08	-0.04 (-0.30)	0.97 (0.81)
Psychiatric disorders	10037175	118	0.31 (0.26,0.38)	0.33 (0.27,0.39)	173.84	-1.62 (-1.87)	0.33 (0.27)
Metabolism and nutrition disorders	10027433	109	0.78 (0.64,0.94)	0.78 (0.65,0.94)	6.87	-0.36 (-0.63)	0.78 (0.65)
Vascular disorders	10047065	99	0.72 (0.59,0.87)	0.72 (0.59,0.88)	10.85	-0.47 (-0.76)	0.72 (0.59)
Eye disorders	10015919	88	0.68 (0.55,0.84)	0.68 (0.56,0.84)	13.09	-0.55 (-0.85)	0.68 (0.55)
Product issues	10077536	34	0.32 (0.23,0.45)	0.32 (0.23,0.45)	49.47	-1.64 (-2.10)	0.32 (0.23)
Surgical and medical procedures	10042613	34	0.38 (0.27,0.54)	0.39 (0.28,0.54)	33.26	-1.37 (-1.83)	0.39 (0.28)
Neoplasms benign, malignant and unspecified (incl cysts and polyps)	10029104	29	0.17 (0.12,0.24)	0.17 (0.12,0.25)	118.19	-2.54 (-3.02)	0.17 (0.12)
Reproductive system and breast disorders	10038604	22	0.38 (0.25,0.58)	0.39 (0.25,0.59)	21.69	-1.37 (-1.94)	0.39 (0.25)
Social circumstances	10041244	16	0.53 (0.32,0.87)	0.53 (0.33,0.87)	6.66	-0.91 (-1.57)	0.53 (0.33)
Ear and labyrinth disorders	10013993	13	0.46 (0.27,0.80)	0.46 (0.27,0.80)	8.05	-1.11 (-1.82)	0.46 (0.27)

Ranked by case reports.

### 3.3 Signal detection related to PT levels about methimazole

**Figure 4:**
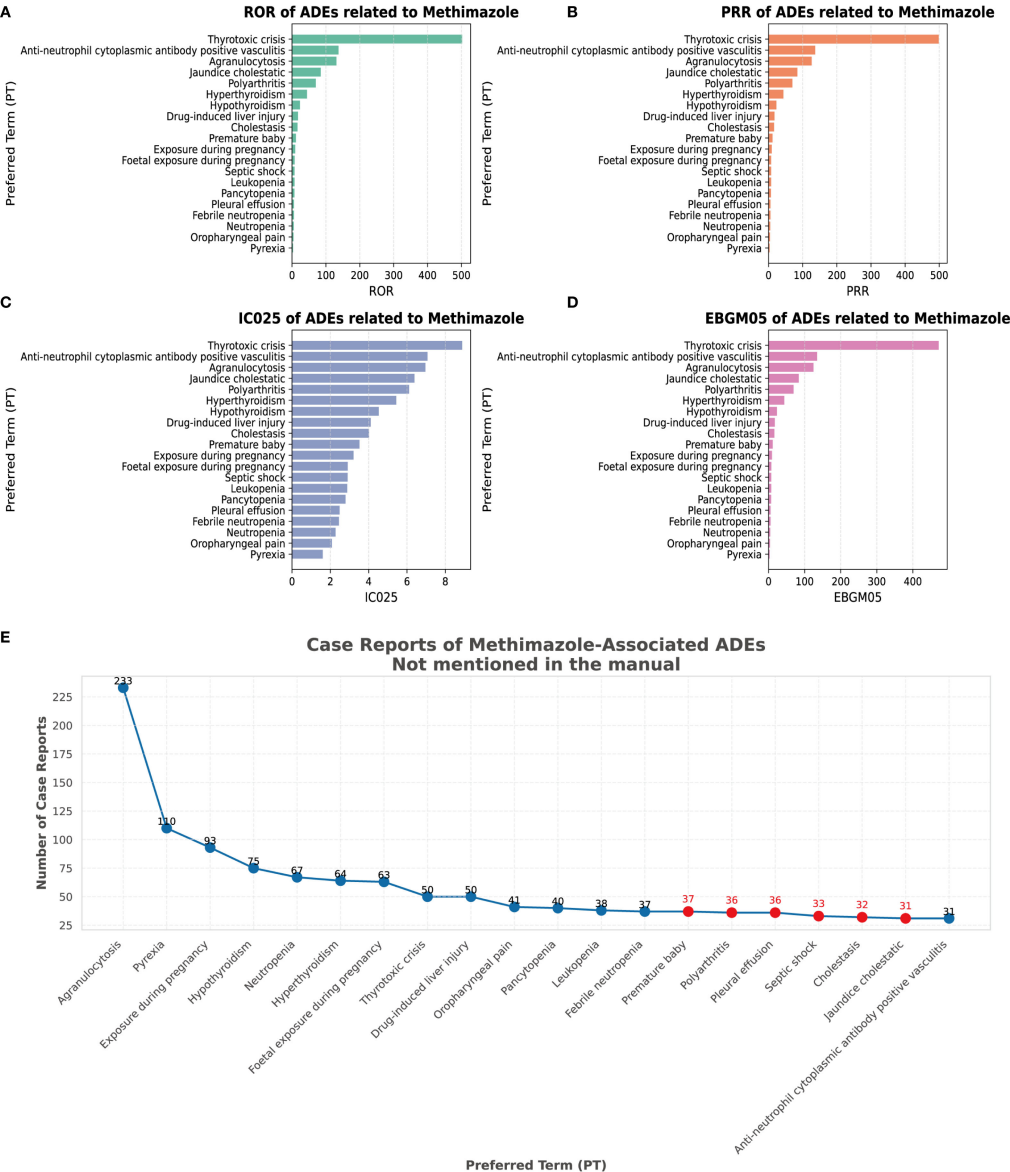
Signal strength at the PT level. **(A)** Satisfy all four algorithms simultaneously and sort them according to ROR. **(B)** Satisfy all four algorithms simultaneously and sort them according to PRR. **(C)** Satisfy all four algorithms simultaneously and sort them according to BCPNN. **(D)** Satisfy all four algorithms simultaneously and sort them according to MGPS. **(E)** The top 20 PTs that simultaneously meet the requirements of all 4 algorithms are sorted by case reports.

We conducted a screening of adverse drug events for methimazole and selected four calculation methods, namely ROR, PRR, BCPNN, and the combined use of MGPS for detecting the detection signals. The threshold was set as follows: a ≥ 3, the lower limit of the 95% ROR confidence interval is greater than 1, PRR ≥ 2, the chi-square value ≥ 4, IC - 2SD > 0, and EBGM05 > 2. Based on the ranking of the frequency of positive signals for the target drug, we selected the top 20 preferred terms. A total of 194 PTs met the criteria across all four calculation methods. Among these reports, the most frequently reported AE was agranulocytosis, with a total of 233 cases. The second most frequently reported adverse event was pyrexia, with a total of 110 cases. The third most frequently reported AE was exposure during pregnancy, with a total of 93 cases. The most common AE was hypothyroidism, with a total of 75 cases. The fourth-ranked AE was hypothyroidism, with a total of 75 cases. The fifth-ranked AE was neutropenia, with a total of 67 cases. **Figures 4A–D** respectively present the ranking of AEs under the four algorithms: ROR, PRR, IC025, and EBGM05. The above AEs are detailed in the instructions of this drug. It should be noted that, in addition to the AEs explicitly mentioned in the instructions of methimazole, this study also identified several common AEs, such as premature baby, polyarthritis, pleural effusion, septic shock, cholestasis, and jaundice cholestatic (**Table 3** **) (** **Figure 4E**). These need to be particularly noted in clinical settings.

**Table 3:** Top 20 signal strength of adverse events at the preferred term (PT) level ranked by reports.

System organ class (SOC)	Preferred term (PT)	Case	ROR (95% CI)	PRR (Chi-Square)	IC (IC-2SD)	EBGM (EBGM05)
Blood and lymphatic system disorders	Agranulocytosis	233	130.96 (114.80-149.39)	126.26 (28551.1)	6.96 (6.15)	124.48 (109.12)
General disorders and administration site conditions	Pyrexia	110	3.07 (2.54-3.71)	3.04 (150.94)	1.60 (1.30)	3.03 (2.51)
Injury, poisoning and procedural complications	Exposure during pregnancy	93	9.41 (7.66-11.54)	9.28 (687.83)	3.21 (2.79)	9.28 (7.56)
Endocrine disorders	Hypothyroidism	75	23.38 (18.62-29.37)	23.12 (1583.98)	4.53 (3.83)	23.06 (18.36)
Blood and lymphatic system disorders	Neutropenia	67	4.85 (3.82-6.18)	4.81 (202.77)	2.27 (1.84)	4.81 (3.78)
Endocrine disorders	Hyperthyroidism	64	43.97 (34.35-56.28)	43.55 (2647.81)	5.44 (4.35)	43.33 (33.86)
Injury, poisoning and procedural complications	Foetal exposure during pregnancy	63	7.60 (5.93-9.74)	7.54 (357.33)	2.91 (2.41)	7.53 (5.88)
Endocrine disorders	Thyrotoxic crisis	50	502.31 (377.35-668.64)	498.42 (23481.2)	8.88 (5.11)	471.56 (354.25)
Hepatobiliary disorders	Drug-induced liver injury	50	17.43 (13.19-23.03)	17.30 (766.78)	4.11 (3.30)	17.27 (13.07)
Respiratory, thoracic and mediastinal disorders	Oropharyngeal pain	41	4.26 (3.14-5.80)	4.24 (101.75)	2.08 (1.53)	4.24 (3.12)
Blood and lymphatic system disorders	Pancytopenia	40	6.97 (5.11-9.51)	6.93 (203.06)	2.79 (2.14)	6.93 (5.08)
Blood and lymphatic system disorders	Leukopenia	38	7.39 (5.37-10.17)	7.35 (208.50)	2.88 (2.20)	7.35 (5.34)
Blood and lymphatic system disorders	Febrile neutropenia	37	5.49 (3.97-7.58)	5.46 (134.83)	2.45 (1.82)	5.46 (3.95)
Pregnancy, puerperium and perinatal conditions	Premature baby	37	11.55 (8.36-15.96)	11.49 (354.09)	3.52 (2.70)	11.48 (8.31)
Musculoskeletal and connective tissue disorders	Polyarthritis	36	70.09 (50.45-97.38)	69.70 (2418.74)	6.11 (4.13)	69.16 (49.78)
Respiratory, thoracic and mediastinal disorders	Pleural effusion	36	5.60 (4.04-7.77)	5.58 (135.24)	2.48 (1.83)	5.57 (4.02)
Infections and infestations	Septic shock	33	7.50 (5.32-10.56)	7.46 (184.67)	2.90 (2.15)	7.46 (5.30)
Hepatobiliary disorders	Cholestasis	32	16.30 (11.51-23.07)	16.22 (456.39)	4.02 (2.97)	16.19 (11.44)
Hepatobiliary disorders	Jaundice cholestatic	31	84.81 (59.49-120.91)	84.41 (2530.75)	6.39 (4.03)	83.61 (58.65)
Immune system disorders	Anti-neutrophil cytoplasmic antibody positive vasculitis	31	137.37 (96.26-196.04)	136.72 (4112.25)	7.07 (4.19)	134.63 (94.34)

### 3.4 Subgroup analysis of age and sex

**Figure 5:**
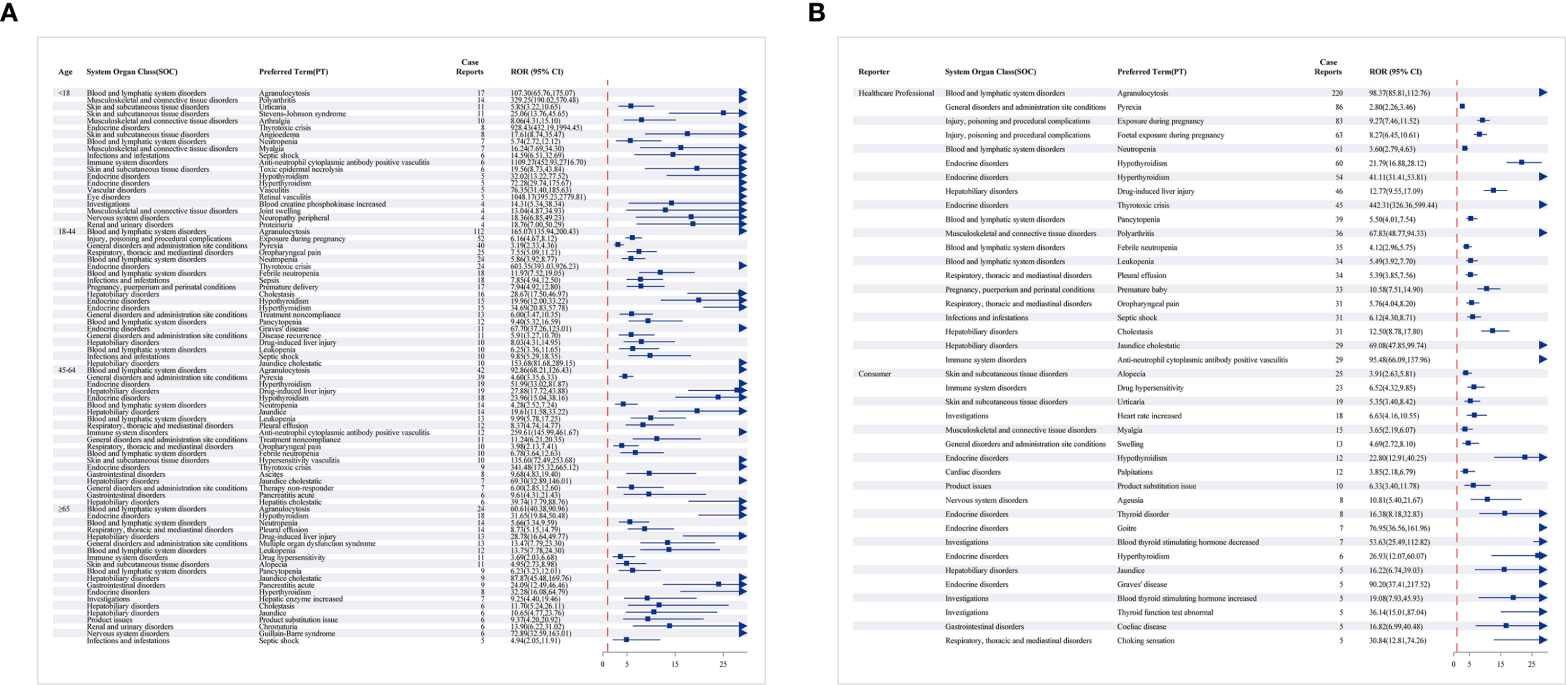
The forest map showing the PT top 20 results for methimazole subgroup analysis. **(A)** Different age groups; **(B)** reporter.

We observed that AE types differed across age groups. Age-stratified subgroup analyses further refined these patterns. Pregnancy exposure and early delivery were reported mainly among individuals aged 18–44 years. Stevens-Johnson Syndrome was observed predominantly among individuals under the age of 18. Guillain-Barré syndrome was observed predominantly among individuals aged 65 years and older. This syndrome was mainly observed in those aged greater than or equal to 65 years old. Agranulocytosis ranked first across all four age groups. Age-stratified analyses revealed distinct age-specific patterns. Among reporters, alopecia, drug hypersensitivity reactions, and urticaria were relatively common; these events were not reported by healthcare professionals (**Figures 5A, B**).

### 3.5 Differential analysis of FAERS data based on age, sex, fatal, and serious

**Figure 6:**
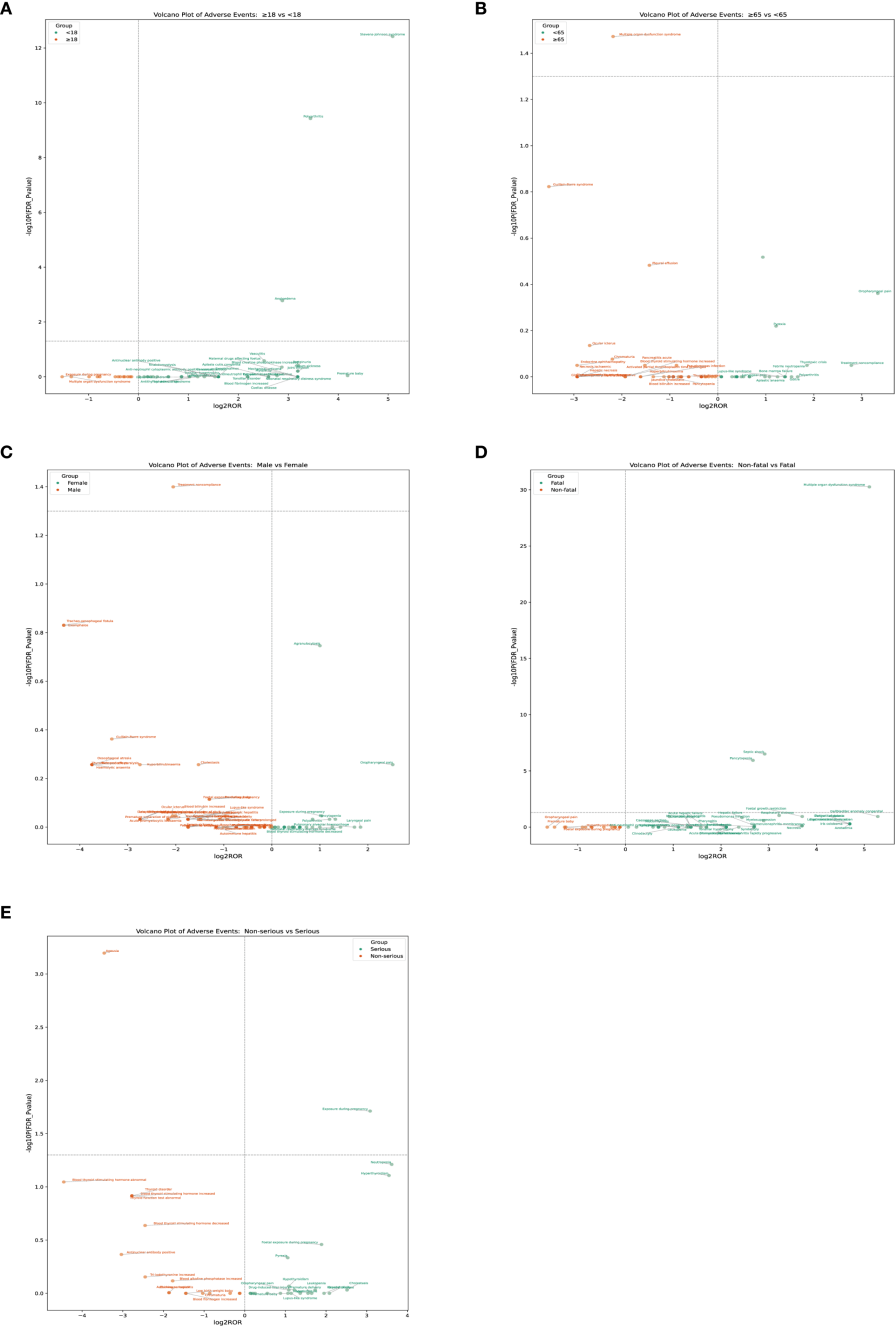
The PT results for methimazole different analysis by age, sex, fatality, and serious. **(A)** Age: adolescents and non-adolescents **(B)** Age: elderly and non-elderly; **(C)** Sex **(D)** Fatal **(E)** Serious.

To investigate potential differences in the occurrence of adverse events across age groups, we compared the reporting patterns of adverse reactions between adolescents and non-adolescents, as well as between the elderly and non-elderly. In the comparison between adolescents and non-adolescents, the incidence rates of angioedema, polyarthritis, and Stevens–Johnson syndrome were significantly higher among adolescents, and these differences were statistically significant (**Figure 6A**). Teenagers may be at a higher risk of certain adverse reactions and therefore require special monitoring and prevention. In the comparison between the non-elderly group and the elderly group, the incidence of multiple organ dysfunction in the elderly group was significantly higher, demonstrating a significant statistical difference (**Figure 6B**). The analysis of gender differences shows that the probability of male patients not complying with the treatment is higher (**Figure 6C**). The analysis of the differences between fatal and non-fatal events indicates that fatal events are mainly concentrated in the upper right quadrant. The most significant signals include multiple organ dysfunction syndrome, septic shock, and pancytopenia (**Figure 6D**). The group with severe illness had a higher incidence of exposure during pregnancy, while in the group with milder illness, the incidence of ageusia was higher, and there was a statistically significant difference (**Figure 6E** **) (** **Table 4**).

**Table 4:** Differential analysis of FAERS data based on age, sex, fatal, and serious.

System organ class (SOC)	Preferred term (PT)	log2ROR	-log10P(FDR_Pvalue)	Group
Skin and subcutaneous tissue disorders	Stevens-Johnson syndrome	5.09	12.42	Age (≥18 vs <18)
Skin and subcutaneous tissue disorders	Angioedema	2.88	2.78	Age (≥18 vs <18)
Musculoskeletal and connective tissue disorders	Polyarthritis	9.28	9.43	Age (≥18 vs <18)
General disorders and administration site conditions	Multiple organ dysfunction syndrome	-2.18	1.47	Age (≥65 vs <65)
General disorders and administration site conditions	Treatment noncompliance	-2.05	1.40	Male vs Female
General disorders and administration site conditions	Multiple organ dysfunction syndrome	5.12	30.26	Non-fatal vs Fatal
Nervous system disorders	Ageusia	-3.46	3.20	Non-serious vs Serious
Exposures associated with pregnancy, delivery and lactation	Exposure during pregnancy	3.08	1.71	Non-serious vs Serious

### 3.6 Time-to-onset and Weibull distribution analysis of AEs based on methimazole

**Figure 7:**
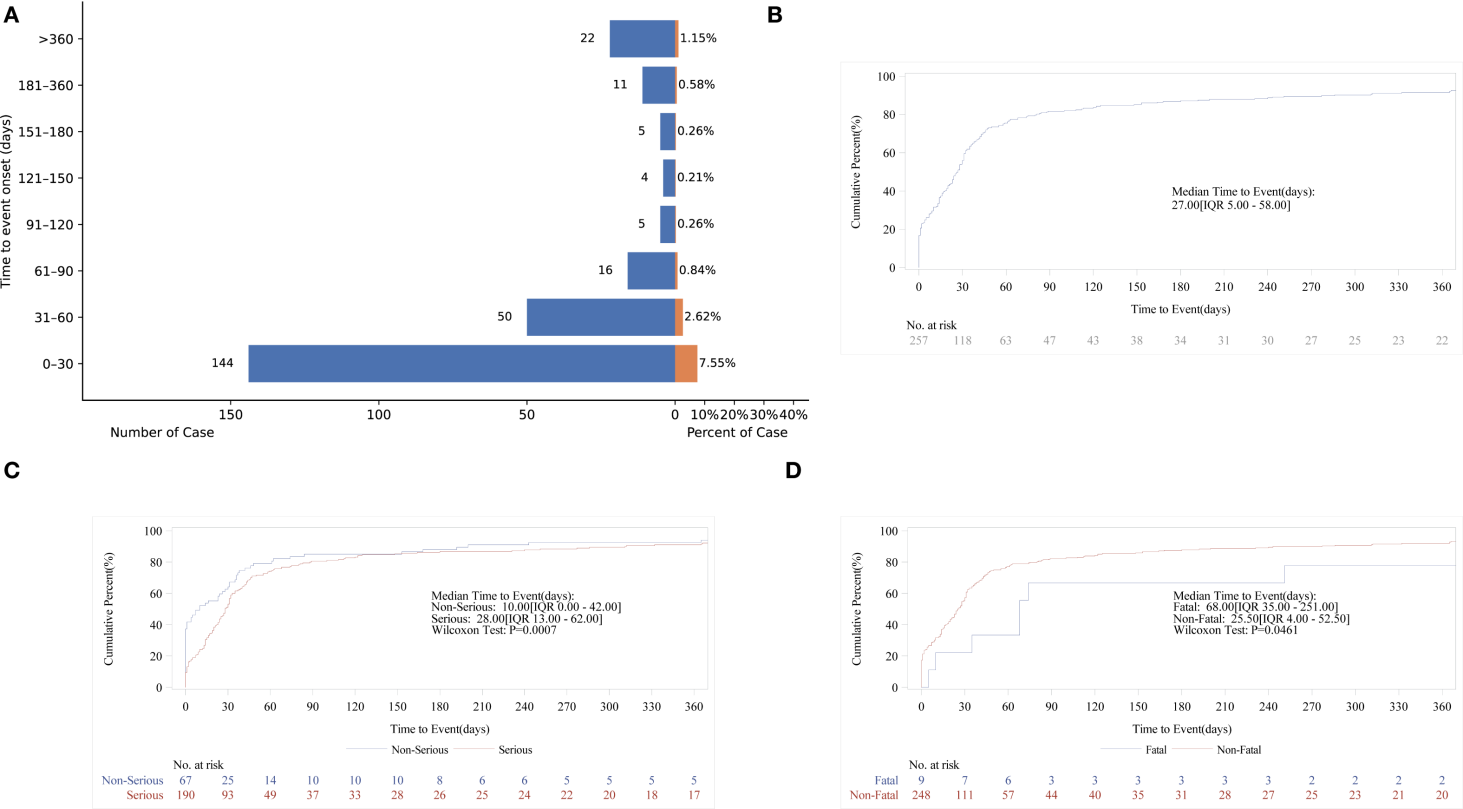
Time-to-onset of methimazole-associated adverse events. **(A)** Bar chart illustrating the distribution of AE onset times following methimazole administration; **(B)** Cumulative incidence curve showing the time-dependent accumulation of AE reports; **(C)** Kaplan–Meier survival curve comparing the onset times of serious versus non-serious AEs; **(D)** Kaplan–Meier survival curve depicting the onset timing of fatal versus non-fatal events.

Time of AE occurrence-medication date not included unspecified was mostly clustered in the range of 0–30 days (86.53%), then 31–60 days (n = 50, 19.46%), and >360 days (n = 22, 8.56%) (**Figure 7A**). The above findings remind us that during the first month of medication, we should pay particular attention to whether AEs occur in the patients. Interestingly, AE can occur at any time within one year after taking methimazole. Subsequent subgroup analysis revealed that the median AE occurrence time-medication date was 27 days (**Figure 7B**). It is indicated that the majority of patients experienced adverse events 27 days prior to the study. The confidence interval (IQR) for the median time was 5.00 days to 58.00 days. At 0 days, 257 individuals were at risk, and at 360 days, 22 individuals were at risk. In the analysis of outcomes following serious outcome events (**Figure 7C**), the median onset time for non-severe adverse events was 10 days (IQR: 0–42 days), whereas severe adverse events had a median onset time of 28 days (IQR: 13–62 days). The temporal distribution of adverse events reveals a severity-dependent pattern, whereby non-serious events predominantly occur early, while serious events emerge later. This delay in onset may reflect underlying pathophysiological processes that require longer time to manifest as severe clinical outcomes. In the fatal outcome group (**Figure 7D**), fatal cases exhibited a median onset time of 68 days (IQR: 35–251 days), substantially later than the 25.5 days (IQR: 4–52.5 days) observed in non-fatal cases. This temporal distinction suggests that fatal events may result from prolonged disease progression or gradual physiological decompensation, requiring extended time to manifest. In contrast, non-fatal events tend to occur earlier and may be amenable to prevention through timely risk stratification and early intervention (**Table 5**).

**Table 5:** Time-to-onset analysis using the Weibull distribution test. .

Weibull distribution						
Cases	TTO (days)	Scale parameter		Shape parameter		Failure type
N	median(IQR)	α	95% CI	β	95% CI	
257	27.00(5.00,58.00)	88.94	68.35 - 115.72	0.54	0.49 - 0.59	Early failure

TTO, time to onset; CI, confidence interval; IQR, interquartile range.

## 4 Discussion

**The demographic pattern of methimazole adverse-event reports appears to reflect underlying disease epidemiology and market exposure rather than intrinsic sex or region specific drug toxicity.** Based on the demographic pattern of adverse events with methimazole: first, the higher share of reports from women likely reflects the higher prevalence of hyperthyroidism in women, which matches known patterns. Second, North America and Asia are probably the main markets for this drug, so higher use there may lead to more reported adverse events. The large populations in these regions may also produce more total cases even if the incidence rates are similar. Third, the concentration of reports in the 18eortsrat group is consistent with patterns of hyperthyroidism in that age range. Finally, differences by reporter type may reflect differences in adverse-event awareness, reporting duties, and access to reporting systems across professional groups.

Methimazole shows a strong hematologic toxicity signal in pharmacovigilance data, led by agranulocytosis, pancytopenia, and leukopenia. In our research, we confirmed the aforementioned findings. We believe that these adverse events may also be connected to the mechanism of action of methimazole (26). A case report described a 36-year-old female who was initially diagnosed with Graves’ disease 2 months prior to admission. At the time of diagnosis, her thyroid serology revealed a free T4 level of 3.76 ng/dL (reference range 0.7-1.5 ng/dL), a free T3 level of 15.1 pg/mL (reference range 1.7-3.7 pg/mL), and TSH receptor antibody levels of 31 IU/L (reference range 0.00-1.75 IUnits/L). Subsequently, her endocrinologist initiated treatment with methimazole 20 mg BID and atenolol 25 mg. Two months later, she presented with right axillary and right buttock abscesses, accompanied by symptoms of nausea, dizziness, and fever. She was found to have significant pancytopenia and elevated liver function test results (27). The convergence of disproportionality signals and clinical reports supports a biologically plausible effect of MMI on hematopoiesis. At the same time, spontaneous-report data may over-represent severe early-onset events and lack exposure denominators; intercurrent infection and disease-related hematologic fluctuations in hyperthyroidism can further modulate the apparent risk in FAERS. Our FAERS analysis suggests that MMI may be associated with preterm birth. In pregnant women with hyperthyroidism, propylthiouracil (PTU) is recommended as the first-line treatment during pregnancy, while methimazole (MMI) is rarely chosen due to its association with congenital malformations. Exposure to MMI during early pregnancy has been linked to rare cases of aplasia cutis (a skin and scalp defect), choanal atresia, and esophageal atresia (28, 29). This finding highlights the need for more cautious evaluation when prescribing MMI to pregnant women and consideration of alternative therapies when appropriate. Rare immune-mediated reactions related to antithyroid therapyroida as antithyroid arthritis syndrome (AAS) characterized by myalgia, arthralgia/arthritis, fever, and rashr,lgi been described after MMI initiation. A study from Japan further reported that antithyroid AAS is a rare adverse reaction linked to antithyroid drugs. The clinical manifestations of AAS are complex and often severe. These events typically occur within a certain period after initiation of MMI therapy and include muscle pain, arthralgia, arthritis, fever, and rash caused by the drug. The pathogenesis of AAS remains incompletely understood, but it has been proposed that it may involve immune-mediated hypersensitivity reactions or direct effects of drug metabolites on joint and muscle tissues. All of the AEs related to MMI mentioned above have been disclosed in the drug labeling, where they are described in detail (30). Proposed mechanisms include immune-mediated hypersensitivity and/or reactive metabolite effects on joint and muscle tissues, which provide a coherent explanation for the constellation of musculoskeletal and systemic symptoms.

Pharmacovigilance signals suggest methimazole may be associated with adverse events not captured in current labeling, most notably polyarthritis and pleural effusion, alongside premature birth, septic shock, cholestasis, and cholestatic jaundice. Our study identified several adverse events that have not yet been documented in the official drug labeling. These events included premature birth, polyarthritis, pleural effusion, septic shock, cholestasis, and cholestatic jaundice. Further analysis showed that polyarthritis appeared to be one of the more frequently observed AEs. Clinicians should therefore remain alert to the possibility of polyarthritis when prescribing antithyroid drugs and should promptly consider this diagnosis when relevant clinical symptoms occur. The timing (within weeks of MMI initiation) and systemic features (fever, rash) support an immune-mediated hypersensitivity consistent with AAS. Differences across studies reflect variation in case definitions, ascertainment sources, and whether rates are exposure-adjusted. We also detected a signal for pleural effusion. A published case report also described a 67-year-old woman who had recently been diagnosed with Graves’ disease and was receiving MMI at a daily dose of 20 mg. Upon admission, she presented with dyspnea and new-onset atrial fibrillation with a rapid ventricular rate. Chest radiography revealed a unilateral right-sided pleural effusion, and thoracentesis confirmed transudative characteristics of the pleural fluid. When combined with our analysis of the adverse event reporting database, these findings suggest that pleural effusion may represent a potential adverse reaction associated with MMI. Clinicians should therefore maintain a high index of suspicion for pleural effusion when prescribing MMI and should consider routine chest imaging follow-up to ensure early detection and to reduce the risk of life-threatening complications (31). Immune-mediated serositis remains a plausible pathway, but confounding by comorbid conditions and missing exposure denominators in spontaneous-report systems can inflate apparent disproportionality. Clinicians should maintain a high index of suspicion for pleural effusion when new respiratory symptoms emerge during MMI therapy and pursue targeted imaging when clinically indicated. In our study, adverse events linked to MMI included premature birth, polyarthritis, pleural effusion, septic shock, and cholestasis. The underlying mechanisms responsible for these AEs remain poorly understood, as no definitive explanations have been reported in the current literature. Continued investigation by other researchers will be essential to clarify these mechanisms and guide safer clinical use of MMI in the future.

Subgroup analyses revealed distinct age, sex, and severity specific risk patterns under methimazole (MMI) exposure. In our study, AEs associated with methimazole included premature birth, polyarthritis, pleural effusion, septic shock, and cholestasis. However, the underlying mechanisms of these reactions remain unclear, as no specific reports or studies have yet addressed them. We hope that future research will further explore these areas as scientific knowledge advances. The differential analysis revealed several noteworthy patterns. When adolescents were compared with non-adolescents, the incidence of angioedema, polyarthritis, and Stevens–Johnson syndrome was found to be significantly higher among adolescents. This difference may reflect the unique characteristics of the adolescent immune system as well as variations in drug exposure. Because adolescents appear to be more susceptible to immune-mediated adverse reactions, clinicians should apply closer monitoring when MMI is prescribed in this age group. In the comparison between elderly and non-elderly patients, the elderly group showed a markedly increased risk of multiple organ dysfunction. This finding could be related to the reduced organ reserve, the presence of comorbidities, and diminished drug metabolism that are common in elderly patients. For these individuals, closer and ongoing assessment of organ function is recommended to detect early signs of dysfunction and to reduce the likelihood of serious outcomes. Sex-based differences were also noted. Male patients were more likely to demonstrate poor treatment adherence, suggesting the need for targeted adherence education and closer follow-up for male patients receiving MMI. These measures may help reduce treatment interruptions or incorrect use of medication. Finally, fatal events differed substantially from non-fatal events. Multiple organ dysfunction syndrome, septic shock, and pancytopenia were the most frequent clinical presentations in fatal cases. These conditions often advance rapidly and are associated with poor outcomes, underscoring the need for early recognition and intervention. The analysis also showed that drug exposure during pregnancy was more common in the group with severe disease, whereas loss of taste was observed more frequently in the less severe group, and both differences were statistically significant. These findings suggest that drug exposure during pregnancy may contribute to more serious adverse outcomes and should be further investigated in future studies. Overall, we observed immune-mediated adverse events in different subgroups. Future studies should clearly define the subgroups (such as age stratification, elderly criteria, gender) to distinguish the effects of the drug from the background of the disease and reporting bias.

Methimazole associated AEs concentrate within the first month of therapy, making early detection and intervention critical. This early aggregation phenomenon is consistent with previous studies, especially the occurrence of granulocyte deficiency and other immune-mediated reactions which typically occur several weeks to several months after the medication is administered. When examining the timing of AEs, our findings showed that the majority occurred within the first month of starting methimazole treatment. Detecting these methimazole-related events as early as possible is essential because they have the potential to be life-threatening. This large-sample, real-world observational study has several limitations that must be recognized. First, reports of AEs and medication errors were submitted on a voluntary basis by pharmaceutical companies, healthcare professionals, and patients, which may have led to incomplete data or reporting bias. Second, the submitted reports were not required to demonstrate a definite causal relationship between the drug and the event. Therefore, these findings should be interpreted as safety signals rather than definitive measures of risk. Further prospective clinical trials with larger cohorts and longer follow-up periods are needed to confirm these observations.

## 5 Conclusion

Methimazole remains a key therapeutic option in the management of hyperthyroidism. While its ability to effectively lower serum thyroid hormone concentrations is well established, it is essential to implement thorough monitoring strategies to detect and address potential adverse events that may occur during treatment. Through a systematic evaluation, this study identified that, beyond the adverse events commonly recognized, particular vigilance should be directed toward premature birth, polyarthritis, pleural effusion, septic shock, and cholestasis. In addition, the spectrum and frequency of adverse events differed among specific patient subgroups, suggesting that certain populations may carry distinct risk profiles. These observations highlight the critical need for individualized monitoring and risk assessment when methimazole is prescribed, thereby enhancing patient safety and informing clinical decisions. Collectively, the present findings provide meaningful insights for healthcare professionals, patients, and other stakeholders, contributing to a more comprehensive understanding of the safety profile of methimazole in real-world clinical practice and supporting future refinements in risk evaluation and management.

## Data Availability

The original contributions presented in the study are included in the article/**Supplementary Material**. Further inquiries can be directed to the corresponding author.

## Supplementary material

Supplementary Table 1 Calculation table.
