# Supplementary material for: Adverse events of the thyroid peroxidase inhibitor methimazole in the treatment of hyperthyroidism: a comprehensive analysis from the first quarter of 2004 to the first quarter of 2025
Source: Front Endocrinol (Lausanne). 2025 Oct 1;16:1680281. doi: 10.3389/fendo.2025.1680281 (PMC12520890; doi:10.3389/fendo.2025.1680281)
Supplement: Supplementary Table 1 — Calculation table. [file Table1.docx]

|  | Thiamazole | Non-Thiamazole |  |
| --- | --- | --- | --- |
| Target AEs | a | b | a+b |
| No-target AEs | c | d | c+d |
|  | a+c | b+d | N=a+b+c+d |

**Supplement Table1**. Calculation table.

[I] ROR algorithm

| Method | Calculation formula | Threshold value |
| --- | --- | --- |
| ROR | 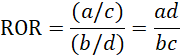  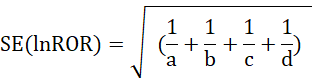  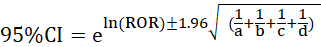 | a≥3 and 95% CI (lower limit) ＞ 1 |

[II] MHRA algorithm

| Method | Calculation formula | Threshold value |
| --- | --- | --- |
| MHRA | 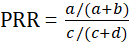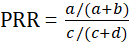  SE(lnPRR)=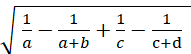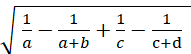  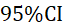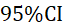=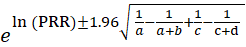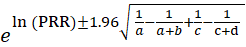  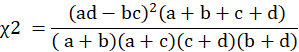 | a≥3 and PRR≥2 and X^2^≥4 |

[III] BCPNN algorithm

| Method | Calculation formula | Threshold value |
| --- | --- | --- |
| BCPNN | IC=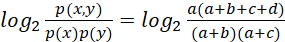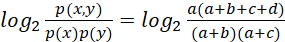  E(IC)=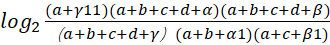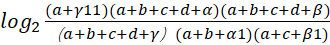  V(IC)=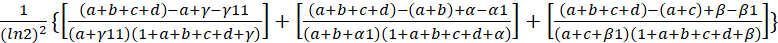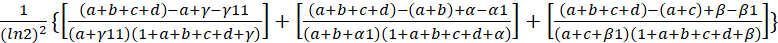  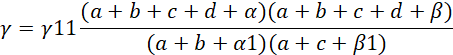  *IC-2SD=E(IC)-2*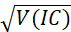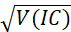  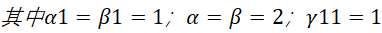 | (-)：IC-2SD ≤0；  (+)：0＜IC-2SD ≤1.5；  (++)：1.5＜IC-2SD ≤3；  (+++)：IC-2SD ＞3 |

[IV] MGPS algorithm

| Method | Calculation formula | Threshold value |
| --- | --- | --- |
| MGPS | 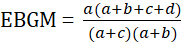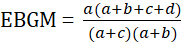  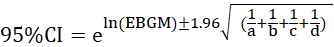 | EBGM05>2 |

Note:

*ROR*, reporting odds ratio;

*CI*, confidence interval;

*PRR*, Proportional reporting ratio;

*χ^2^*, chi-squared;

*BCPNN*, bayesian confidence propagation neural network;

*IC*, information component;

*IC025*, the lower limit of the 95% two-sided CI of the IC；

*EBGM*, Empiric Bayes geometric mean;

*EBGM05,* the lower 95% one-sided CI of EBGM.
